# A pathway analysis applied to Genetic Analysis Workshop 16 genome-wide rheumatoid arthritis data

**DOI:** 10.1186/1753-6561-3-s7-s91

**Published:** 2009-12-15

**Authors:** David H Ballard, Chatchawit Aporntewan, Ji Young Lee, Joon Sang Lee, Zheyang Wu, Hongyu Zhao

**Affiliations:** 1 Program in Computational Biology and Bioinformatics, Yale University, 300 George Street, Suite 503, New Haven, Connecticut 06511, USA; 2 Department of Psychiatry, Yale University, 300 George Street, Suite 503, New Haven, Connecticut 06511, USA; 3 Keck Laboratory, Yale University, 300 George Street, Suite 503, New Haven, Connecticut 06511, USA; 4 Department of Epidemiology and Public Health, Yale University, 300 George Street, Suite 503, New Haven, Connecticut 06511, USA; 5 Department of Genetics, Yale University, 300 George Street, Suite 503, New Haven, Connecticut 06511, USA

## Abstract

The identification of several hundred genomic regions affecting disease risk has proven the ability of genome-wide association studies have proven their ability to identify genetic contributors to disease. Currently, single-nucleotide polymorphism (SNP) association analysis is the most widely used method of genome-wide association data, but recent research shows that multi-marker tests of association may provide greater power, especially when more than one mutation is present within a gene and the mutations are in low linkage disequilibrium with each other. Here we use a multi-marker association test based on regression to SNPs located within known genes to obtain a gene-level score of association. We then perform pathway analysis using this score as a measure of gene importance. We use two tests of pathway enrichment - a binomial test and a random set method. By utilizing publicly available gene and pathway information, we identify B cell, cytokine and inflammation response, and antigen presentation pathways as being associated with rheumatoid arthritis. These results confirm known biological mechanisms for auto-immunity disorders, of which rheumatoid arthritis is one.

## Background

Rheumatoid arthritis (RA) is a complex genetic autoimmune disease that is characterized by pain and swelling in the joints of the body [1]. According to the National Institute of Arthritis and Musculoskeletal and Skin Diseases [2], about 1.3 million adults in the U.S. suffer from RA. While the exact cause of the disease is unknown, it is believed that it is due to three basic factors: genetics, environment, and hormones. In order to identify the genetic factors involved, researchers have performed many linkage and association studies. These studies have implicated several genetic contributors, many of which have not been replicated. One region that has been replicated is the association of the major histocompatibility complex (MHC) on chromosome 6, with HLA_DRB1 alleles consistently found to be associated with RA [3]. Other genetic contributors that have more recently been identified and verified, primarily thanks to the increased power and localization provided by genome-wide association studies (GWAS), are *PTPN22 *and *TRAF1-C5 *[4].

GWAS are best suited for identifying common alleles with small to moderate effects on complex disease risks. To date, several research groups have undertaken this approach to identify the genetic components of RA. Here we concentrate on the GWAS performed by Plenge et al. [4]. Their study successfully combined the interrogation of hundreds of thousands of SNPs, a well defined clinical outcome, and high-quality control standards to identify the association of *TRAF1-C5 *and confirm the association of *PTPN22 *and *MHC*, each attaining a significance level < 5 × 10^-8^. The ability of the study to replicate known results and to identify new associations highlights the good design of the study, and therefore may provide power for additional findings with further, more refined analysis. Here, we will apply known biological information to the analysis, namely gene and pathway annotation.

Given the success of gene and pathway analysis of gene expression data [5], Wang et al. applied similar methodology to a GWAS on Parkinson's disease. Their method used the maximum test statistic of all SNPs located near or on a gene as the gene-level score. They then modified the gene set enrichment analysis (GSEA) algorithm [6], previously used for gene expression studies, to identify enriched pathways that are associated with the disease. GSEA applies a windowed Kolmogorov-Smirnov test to a ranked list of genes (ordered by the association test above) to determine whether a predefined set of genes is enriched compared with a random selection of genes. Using similar pathway information but different methods, we attempted to identify pathways responsible for RA. We employed regression as a test for multi-marker association to generate a gene-level score, and the binomial approximation of Fisher's exact test to identify enrichment of biological pathways. Regression has been shown to be a more powerful method than single-SNP (single-nucleotide polymorphism) analysis due to its ability to exploit linkage disequilibrium [7] and will be computational faster than using the best-scoring SNP followed by permutation to obtain a *p*-value. Lastly, we implemented a random set gene enrichment method [8] and compared the pathways identified by both methods.

## Methods

The first step in our method is to calculate a gene-level score. In order to perform the linear regression, we converted the text genotypes into a numeric genotype score (0, 1, or 2) based on the count of the minor allele. SNPs with a minor allele frequency < 0.01 or a *p*-value < 0.001 for Hardy-Weinberg equilibrium were excluded from the analysis. Additionally, only SNPs and subjects who had <10% missing values were included. Our analysis included 528,719 autosomal SNPs for 2,002 subjects (862 cases and 1140 controls) following the quality control filtering. The SNP were then mapped to known genes. Gene information (gene symbol, chromosome, start position, and end position) present in the Refseq annotation was downloaded from NCBI [9] for build 38. SNPs were considered to be on a gene by using the SNP annotation file provided by Plenge et al. [4] for Genetic Analysis Workshop 16. For each gene, we queried the SNP genotypes associated with the gene and regressed case-control status onto the SNP genotype values. The gene score is the *p*-value of this multiple regression. We obtained gene-level scores for 15,107 genes.

In order to determine the enrichment of pathways, we gathered publicly available pathway information from three public databases: KEGG [10], GenMAPP [11], and BioCarta [12]. The pathways were downloaded from each database and aggregated. There were a total of 564 pathways comprising a total of 5,464 unique genes. Pathways ranged in size from 1 to 399 genes, had a median number of 28 genes, and averaged 47 genes per pathway. We did not require there to be a minimum number of genes in order to be considered a pathway.

Pathway enrichment was determined using two different methods, a binomial test and a random set method. The binomial test is an approximation to the Fisher's exact test, in which the *p*-value of enrichment is determined from the hypergeometric distribution. This approximation has been shown to be robust and dependable in previous studies of enrichment using gene expression data [13]. The probability of success for the binomial distribution was calculated by setting a threshold level below which a gene is considered 'significant', then calculating the fraction of all gene scores below this threshold level. The significance of each pathway was calculated by computing the fraction of genes within the pathway below the threshold used above, the size of the pathway, and the probability of success determined by considering all genes. In order to evaluate the impact of the arbitrary threshold of significance chosen, we used three different threshold of significance (0.01, 0.1, and 0.2) and compared their results.

The second method, the random set method, computes a test statistic equal to the negative of the sum of the log(p-values) for each gene in the pathway [4]. The significance of the pathway is determined by permuting the gene scores, then recalculating the test statistic for each pathway. This permutation procedure was performed 10,000 times, and the p-value of enrichment was set equal to the number of random sets based on permutation that had a test statistic less than the observed original data. This statistic will be more influenced by better scoring genes than the binomial method. For example, if a pathway contains one very significant gene (a multiple regression p-value near zero) or many genes with gene scores that are slightly above a significance threshold, then the pathway is more likely to be deemed significant using this method than the binomial. In the binomial setting, it is necessary to have several genes deemed significant below the set threshold in order to be significant. We anticipate that the random set method may identify more significant pathways than the binomial method, especially when the MHC region is included. We performed the pathway analysis including and excluding the MHC region in order to compare the ability of these methods to identify RA-related pathways.

Due to the inherent correlation among genes due to linkage disequilibrium, the pathway enrichment *p*-values are not independent and require an estimate of the false-discovery rate. We calculated a measure of the false-discovery rate by performing a permutation procedure as follows. Gene scores were recalculated 1000 times using permuted disease status. For each set of gene scores, the pathway analysis (binomial at each threshold and the random set method) was performed using all genes, and using non-MHC genes. The false-discovery rate was obtained by counting the number of pathways with a *p*-value less than that obtained using the real data for each iteration and taking the average over the permutations. We use this value, approximately equal to the false-discovery rate, to determine the significance of each pathway.

## Results

**Figure 1:**
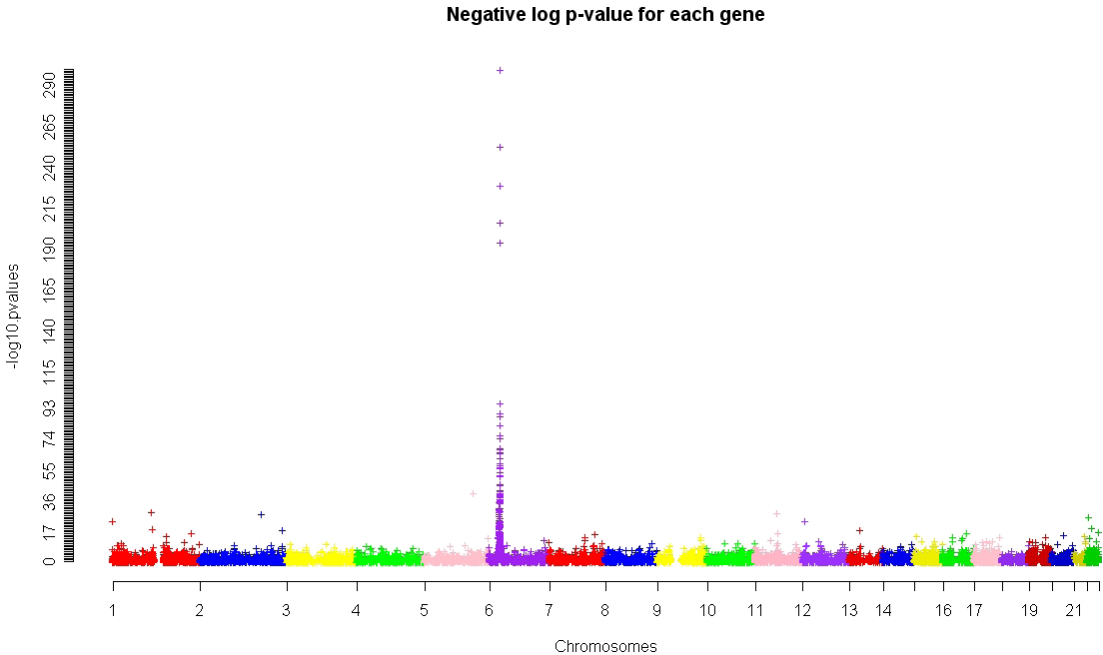
**Plot of all gene scores relative to gene position**. Plot of -log(*p*-value) of the multiple regression *p*-value for each gene at its relative position in the genome.

**Figure 2:**
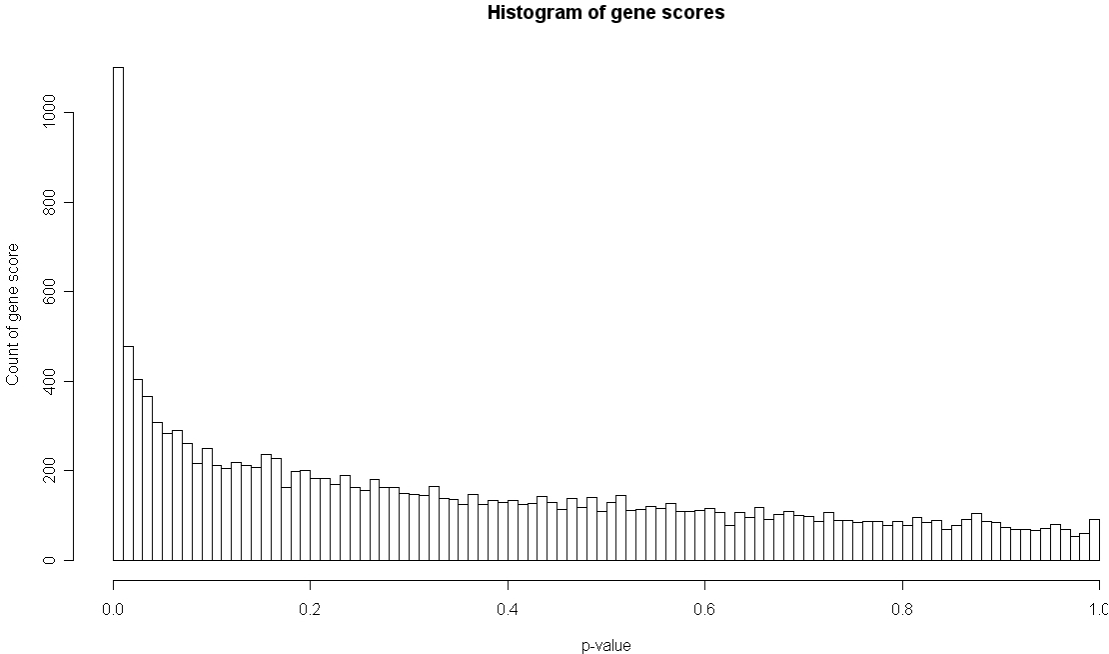
**Histogram of gene score enrichment**. Histogram of gene scores (multiple regression *p*-values) for genes containing SNPs in the study.

The negative log of the gene scores relative to their genomic location are shown in Figure 1. There are a total of 15,107 genes. The highest scoring genes were *HLA-DRB1 *and *HLA-DRA*: both had regressions resulting in a *p*-value of approximately 0. The log values of these *p*-values were set to 300 for Figure 1. The top 100 genes were primarily in the 6p21 region, which represents the MHC region. The top-scoring gene outside of the MHC region was *CDC25C *on chromosome 5. Of the five known genes associated with RA, *HLA-DRB1 *tied for the highest rank, *PTPN22 *ranked 83^rd^, *TRAF1 *ranked 119^th^, C5 ranked 631^st^, and *STAT4 *ranked 3,182^nd^. All but one of these genes had a high-scoring SNP within the gene. *HLA-DRB1 *had the highest single-SNP association score of all SNPs tested; it had a chi-square test statistic of 500.4. The highest single-SNP chi-square values for the other known genes are: 42.97 (*PTPN22*), 30.31 (*TRAF1*), 32.10 (*C5*), and 6.30 (*STAT4*). Many genes have been reported to have an association to RA in different studies, but their true association status is not definite. Among them are *IKBL*, *BAT2*, *CTLA4*, *PADI4*, *TNFAIP3*, *CD40*, *BDKR1*, and *CCL1*. The ranks of these potential RA genes by gene score are 145, 19, 421, 1550, 666, 1951, 2348, and 69, respectively. Figure 2 shows the overall distribution of gene scores. We did not observe larger genes having more significant gene scores. A regression of the count of SNPs within the gene (we assumed larger genes would have more SNPs contained within their boundaries) onto the gene score resulted in a *p*-value of 0.18.

Genes assigned to pathways do not equally represent the genomic regions. Specifically, the genomic regions containing the highest gene scores differed between those identified overall and those located in pathways; however, the gene region with the highest number of genes located within it was the same for both the pathway regions and overall regions - 6p21.3 (the MHC region). At the 0.01 threshold level, five of the top ten genomic regions were the same. Six of the top ten regions were the same at the 0.1 threshold level, and seven out of ten at the 0.2 threshold level. Table 1 lists the top ten regions represented when considering all genes compared with using only genes within the pathways. The regions do vary by the threshold chosen, with only 6p21.3 remaining at the top for each threshold. This region (MHC) is known to be associated with RA, and also represents the only region definitely linked to RA in family-based linkage studies [14]. Other regions that have shown suggestive linkage in past family studies (1p13, 1q41-43, 6q16, 16p, and 18q) are not represented. In order to assess the impact of genomic region 6p21.3 on the pathway results, we removed all 156 genes located in the region and recalculated the pathway scores using the three thresholds. Despite the lack of spatial equality of the genome in the pathway tests, the percent of genes used by the binomial test were consistent across the different thresholds. Specifically, there were 1101 out of the total 15,107 genes with a gene score < 0.01; 352 of these genes were located in the pathway annotation. Fifty-four of the genes were located in 6p21.3. Using the threshold of 0.1, 3957 genes had gene scores below 0.1, of which 1258 were located in pathways. When the threshold was set to 0.2, 6040 genes had scores below the cut-off and 1915 of them were located within the pathway annotation. Hence, approximately 32% of overall genes were consistently located in annotated pathways irrespective of the threshold chosen.

**Table 1:** Top ten genomic regions interrogated by pathways

	Threshold					
	
	0.01		0.1		0.2	
			
Rank	Pathway^a^	Overall^b^	Pathway	Overall	Pathway	Overall
1	6p21.3	6p21.3	6p21.3	6p21.3	6p21.3	6p21.3
2	6p22.1	19p13.3	21q22.3	19p13.3	1q21	19p13.3
3	6p21	6p22.1	5q31	21q22.3	19q13.2	16p13.3
4	13q34	6p21.33	19p13.2	16p13.3	19p13.3	21q22.3
5	16q22.1	16q22.1	16p13.3	19p13.2	16p13.3	19p13.2
6	6p21.31	17q12	11p15.5	11p15.5	21q22.3	14q11.2
7	16p11.2	21q22.3	11q13	14q11.2	19p13.2	1q32.1
8	2q33	2q11.2	19p13.3	17q25.1	19q13.1	19q13.2
9	19q13.4	14q11.2	1q21	11q11	11p15.5	11p15.5
10	1q21	6p21	17p13.1	19q13.2	5q31	11q11

^a^Pathway, only genes within the pathway were considered.

^b^Overall, all genes were considered.

The significant pathways from our analysis are reported in Additional files 1 and 2. The pathways shown had a binomial or random set false-discovery rate estimated from our permutation procedure < 0.01. The top-scoring pathways were consistent across the methods and were related to the immune response, which defines RA. The pathways that were significant at each of the binomial thresholds as well when using the random set method are: BioCarta's Bystander B Cell Activation Pathway, Biocarta's Antigen Dependent B Cell Activation Pathway, KEGG's Antigen Processing and Presentation Pathway, BioCarta's IL 5 Signaling Pathway, BioCarta's Lck and Fyn Tyrosine Kinases in Initiation of TCR Activation Pathway, BioCarta's Human Activation of Csk by cAMP-dependent Protein Kinase Inhibits Signaling Through the T Cell Receptor Pathway, BioCarta's Human Cytokine and Inflammatory Response Pathway, and BioCarta's Humna Th1/Th2 Differentiation Pathway. The genes that make up these pathways are, however, primarily located in the MHC region. The only top pathway not dominated by genes in the MHC region is GenMAPP Human Adipogenesis. After removing the 156 genes located in 6p21.3, some of the previously top-scoring pathways associated with B Cell Activation and Antigen Processing and Presentation were no longer significant when using the binomial test. The only pathway that was significant across the board following the removal of 6p21.3 genes was GenMAPP Human Adipogenesis. Interestingly, even when the MHC region genes were removed, the random set method continued to identify many of the immune-related pathways identified by both methods before the removal of 6p21.3 genes.

As expected, the random set method identified more pathways than the binomial method using all genes and when MHC region genes were removed. The random set method identified 34 pathways as significant compared with 15, 13, and 14 for the binomial when a threshold of 0.01, 0.1, and 0.2 were used, respectively. The same was true when 156 MHC region genes were removed and the pathway significance was assessed again; however, the difference was more dramatic. The random set method identified 50 pathways as significant, while the binomial identified only 2, 6, and 7 for the 0.01, 0.1, and 0.2 thresholds. This change, an increased number found by random set and decreased number identified by the binomial test, reflects how each method determines significance. For the binomial test, the removal of significant genes lessens the probability that a pathway will have genes that are below the set threshold, resulting in fewer pathways being identified. In contrast, the random set method determines significance using only genes that are available. In effect, there is less competition for pathways containing one or two significant genes compared to random sets of genes, and this leads to more pathways classified as significant.

## Discussion

Applying pathway annotation information to the analysis of GWAS data may lead to a better biological understanding of the disease being studied [15]. RA is a systemic autoimmune disease. Our bodies typically initiate an immune response when foreign invaders (antigens) enter our bodies. Antibodies that have been produced by B cells identify antigens; these antibodies have an antigen-binding region and a tail region that binds to receptors on macrophages. Macrophages are built to engulf and destroy these invaders, but they also produce cytokines (proteins used to communicate to other cells that an antigen has been found) like tumor necrosis factor (TNF) and interleukin (IL)-1, and signal the adaptive immune system (T-cells) using MHC proteins that an infection is underway. These MHC class II molecules are used to communicate to helper T-cells the state of a current infection. The type of infection will determine the type of helper T-cell that is activated and hence the kind of cytokines that will be produced. Type 1 helper T-cells (Th1) typically produce IFN-γ, IL-2, and TNF, while Type 2 helper T-cells (Th2) produce IL-4, IL-5, and IL-10. When RA affects someone, the balance in this response is disturbed-the immune response is activated when antibodies recognize self cells as invaders, which then activate macrophages. The inflammation found in RA patients is caused by the abundance of TNF from this immune response. In fact, treatments for RA try to block production of TNF [16].

Risk of RA has both genetic and environmental components. The exact mechanism of RA is unknown, but several genes and pathways have been identified. For example, smoking is a known risk factor. Smoking can cause a post-translational modification of some proteins, which results in a higher binding affinity by *HLA-DRB1 *(A MHC class II molecule) and leads to an exaggerated helper T-cell response [17]. Variants of the gene *PTPN22 *have been shown to lead to a predisposition of autoimmunity by effecting the removal of auto-reactive T-cells [18]. Additionally, the activation of *STAT4 *by IL-12 can bias the production of Th1 and Th17 from virgin T-cells instead of Th2 [18]. The Th1/Th17 inflammatory response leads to an accumulation of the cytokine TNF, which is a signature of RA [18].

Our gene scores corroborate these known results, in particular the association with the MHC region. The top two scoring genes were *HLA-DRA *and *HLA-DRB1*. Alleles of *HLA-DRB1 *have been identified by others to be associated with RA. The shared-epitope hypothesis describes which of the particular MHC class II molecules are associated with RA susceptibility and RA severity [3]. In Figure 2, the enrichment of genes with low *p*-values is apparent. Given this enrichment, we believe that there is relevant biological information on which to capitalize. Other previously reported genes associated with RA did have high ranks in our analysis. For example, *PTPN22 *ranked 83^rd ^and *TRAF1 *ranked 119^th^. *STAT4*, however, did not rank highly, but the authors who provided the data used a combined data set in order to find that association. The highest non-MHC gene was *CDC25C*, which ranked 30^th^. No current research associates *CDC25C *to RA, but it does interact with Lck - a tyrosine kinase involved in TCR activation [19].

The pathway results support the known biological processes involved in RA. Using the 0.01 threshold and sorting by their significance, we found pathways for antigen processing and presentation, B cell activation, T cell activation, and the complement pathway at the top. The approximately 156 genes in the MHC region dominate these pathways. But, each of these pathways is integral to the immune response. Exploring the remaining pathways in Supplemental File 1 [1753-6561-3-S7-S91-S1.pdf], we see several other pathways that relate to other known theories of RA. For example, a case could be made for BioCarta's Human Extrinsic Prothrombin Activation Pathway. Animal studies have shown that cartilage degeneration due to joint immobilization increases the expression of prothrombin gene in chondrocytes [20]. Additionally, findings showing the pro- and anti-inflammatory properties of adipose tissue make GenMAPP's Human Adipogenesis Pathway a likely candidate for RA [21]. Lastly, removing the genes located in the 6p21.3 region did remove the most significant pathways associate with B cell activation, but many other potential pathological pathways remained, specifically the Adipogenesis Pathway. While the cell cycle pathways were not unanimously significant, their presence following the removal of 6p21.3 was striking considering their known role in RA [22].

## Conclusion

Here we have demonstrated the methods and usefulness for applying gene and pathway annotation information to GWAS. We performed a multiple regression of case-control status onto all SNPs located within a gene to generate a gene-level score of association. Regression has shown to be a powerful method of multi-locus association, especially when there is little to no linkage disequilibrium among the SNPs [23]. Our results show an enrichment of associated genes, many of which are known genes associated with the disease. Given this confirmation of available information, we used these gene-level scores as a basis for identifying pathways associated with RA disease status. By using different thresholds in our analysis, we were able to evaluate the genes at different interest levels. Here the results were consistent across different thresholds, and were corroborated by pathways identified by the random set method. This overlap may not be found in every study, and may be due to the strong signal present at MHC. We suspect that by choosing different thresholds, researchers will be able investigate the impact of genes at different association levels. Given that pathway-based analysis is not the first pass researchers will use to identify associations, it does provide a means to mine information present not captured by single-SNP analysis.

## List of abbreviations used

GSEA: Gene set enrichment analysis; GWAS: Genome-wide association studies; IL: Interleukin; MHC: Major histocompatibility complex; RA: Rheumatoid arthritis; SNP: Single-nucleotide polymorphism; Th1 and Th2: Type 1 and 2 helper T-cells; TNF: Tumor necrosis factor.

## Competing interests

The authors declare that they have no competing interests.

## Authors' contributions

DHB and SYL performed data processing. DHB performed analysis. All authors participated in design of the study and synthesis of results. HZ coordinated analysis. All authors read and approved the final manuscript.

## Supplementary Material

Additional file 1 Significant pathways. Click here for file

Additional file 2 Significant pathways when genes located within 6p21.3 are removed. Click here for file
