# Supplementary material for: A pathway analysis applied to Genetic Analysis Workshop 16 genome-wide rheumatoid arthritis data
Source: BMC Proc. 2009 Dec 15;3(Suppl 7):S91. doi: 10.1186/1753-6561-3-s7-s91 (PMC2795995; doi:10.1186/1753-6561-3-s7-s91)
Supplement: Additional file 1 — Significant pathways. [file 1753-6561-3-S7-S91-S1.pdf]

Supplemental File 1 - Significant pathways

Pathway[a]	Provenance	Binomial[b]			Random set
		0.01	0.1	0.2	
Activation of Csk by cAMP-dependent Protein Kinase Inhibits Signaling through the T Cell Receptor Pathway	Biocarta	2.6×10^{-4}	3.7×10^{-3}	9.2×10^{-3}	0
Activation of Src by Protein-tyrosine phosphatase alpha pathway	Biocarta				2.4×10^{-3}
Acute Myocardial Infarction Pathway	Biocarta		6.3×10^{-3}		
Alternative Complement Pathway	Biocarta				0
Antigen Dependent B Cell Activation Pathway	Biocarta	2.4×10^{-4}	2.8×10^{-3}	5.8×10^{-3}	0
B Lymphocyte Cell Surface Molecules Pathway	Biocarta				0
Bystander B Cell Activation Pathway	Biocarta	2.4×10^{-4}	2.8×10^{-3}	5.8×10^{-3}	0
Cdc25 and chk1 Regulatory Pathway in Response to DBA Damage	Biocarta				5.6×10^{-3}

Chaperones modulate interferon Signaling Pathway	Biocarta		7.7×10^{-3}		
Classical Complement Pathway	Biocarta	9.7×10^{-4}			2.0×10^{-3}
Complement Pathway	Biocarta	8.2×10^{-4}			0
Cytokines and Inflammatory Response pathway	Biocarta	2.4×10^{-4}	$3.2 \text{ x } 10^{-3}$	6.1×10^{-3}	0
Extrinsic Prothrombin Activation Pathway	Biocarta		2.8×10^{-3}		
IL 5 Signaling Pathway	Biocarta	2.4×10^{-4}	2.8×10^{-3}	5.8×10^{-3}	0
Lck and Fyn tyrosine kinases in initiation of TCR Activation pathway	Biocarta	2.4×10^{-4}	2.9×10^{-3}	6.4×10^{-3}	0
Lectin Induced Complement Pathway	Biocarta				2.8×10^{-3}
NO2-dependent IL 12 Pathway in NK cells pathway	Biocarta			8.9×10^{-3}	
Pelp1 Modulation of Estrogen Receptor Activity Pathway	Biocarta				8.5×10^{-3}
Presenilin action in Notch and Wnt Signaling Pathway	Biocarta				3.6×10^{-3}
Proteolysis and Signaling Pathway of Notch pathway	Biocarta				0
Ras-Independent pathway in NK cell-mediated cytotoxicity pathway	Biocarta	1.9×10^{-3}			2.0×10^{-3}
Regulation of cell cycle progression by Pik3 Pathway	Biocarta				5.0×10^{-3}

Sonic Hedgehog Receptor Ptc1 Regulates cell cyle Pathway	Biocarta				3.1×10^{-3}
Th1/Th2 Differentiation pathway	Biocarta	2.3×10^{-3}	4.1×10^{-3}	6.5×10^{-3}	0
The Co-Stimulatory Signal During T-cell Activation pathway	Biocarta			8.9×10^{-3}	0
The Role of Eosinophils in the Chemokine Network of Allergy pathway	Biocarta				0
Visceral Fat Deposits and the Metabolic Syndrome pathway	Biocarta				7.5×10^{-3}
Human_Adipogenesis Human	GenMapp	3.3×10^{-4}	5.4×10^{-3}	6.0×10^{-3}	
Genes specific to blood and lymph tissue	GenMapp				0
Human_Proteasome Degradation	GenMapp	2.6×10^{-4}			3.0×10^{-3}
Triacylglyceride Synthesis	GenMapp				9.8×10^{-3}
Cell adhesion molecules (CAMs)	KEGG	2.4×10^{-4}	2.9×10^{-3}		0
Complement and Coagulation Cascades	KEGG				4.4×10^{-3}
Antigen processing and presentation	KEGG	2.4×10^{-4}	2.8×10^{-3}	5.8×10^{-3}	0

ECM Receptor Interaction	KEGG			9.4×10^{-3}
Fatty acid elongation in Mitochondria	KEGG			8.9×10^{-3}
Jak-STAT signaling pathway	KEGG		9.3×10^{-3}	
Monoterpenoid Biosynthesis	KEGG			9.5 x 10^{-3}
Natural killer cell mediated cytotoxicity	KEGG	3.3×10^{-4}	8.8×10^{-3}	0
Notch Signaling	KEGG			3.5×10^{-3}
Prostate cancer	KEGG		8.3×10^{-3}	

[a]Pathways that were identified as significant (estimated false-discovery rate < 0.01). The pathway's estimated false-discovery rate is listed in its respective cell.

[b]The threshold is used as the probability of success.
