# Supplementary material for: A pathway analysis applied to Genetic Analysis Workshop 16 genome-wide rheumatoid arthritis data
Source: BMC Proc. 2009 Dec 15;3(Suppl 7):S91. doi: 10.1186/1753-6561-3-s7-s91 (PMC2795995; doi:10.1186/1753-6561-3-s7-s91)
Supplement: Additional file 2 — Significant pathways when genes located within 6p21.3 are removed. [file 1753-6561-3-S7-S91-S2.pdf]

Supplemental File 2 - Significant pathways when genes located within 6p21.3 are removed

		Binomial[b]			
Pathway[a]	Provenance	0.01	0.1	0.2	Random set
Activation of Csk by cAMP-dependent Protein Kinase Inhibits Signaling through the T Cell Receptor pathway	Biocarta				8.9×10^{-3}
Activation of Src by Protein-tyrosine phosphatase alpha pathway	Biocarta				2.7×10^{-4}
Acute Myocardial Infarction pathway	Biocarta		5.8×10^{-3}		3.2×10^{-3}
Antigen Dependent B Cell Activation pathway	Biocarta				0
Bystander B Cell Activation pathway	Biocarta				0
CD40L Signaling Pathway	Biocarta				2.8×10^{-3}
Cdc25 and chk1 Regulatory pathway in Response to DNA Damage	Biocarta				1.7×10^{-3}
Cell Cycle: G1/S Check Point pathway	Biocarta				0
Cell Cycle: G2/M Checkpoint pathway	Biocarta				6.2×10^{-5}
Chaperones modulate interferon signalling pathway	Biocarta		9.3×10^{-3}		7.3×10^{-4}
Classical Complement Pathway	Biocarta	3.5×10^{-3}			
Co-Stimulatory Signal During T-cell	Biocarta				9.5×10^{-5}

Activation Pathway				
Cyclins and Cell Cycle Regulation pathway	Biocarta			2.3×10^{-5}
E2F1 Destruction Pathway pathway	Biocarta			6.3×10^{-3}
EPO Signaling Pathway pathway	Biocarta			5.1×10^{-3}
Erythropoietin Mediated Neuroprotection through NK-kB Pathway	Biocarta			2.0×10^{-3}
Extrinsic Prothrombin Activation Pathway	Biocarta	7.5×10^{-3}		7.8×10^{-3}
Gamma-aminobutyric Acid Receptor Life Cycle Pathway	Biocarta			3.8×10^{-3}
Growth Hormone Signaling Pathway	Biocarta			6.9×10^{-3}
How Progesterone Initiates Oocyte Membrane pathway	Biocarta			7.3×10^{-5}
IFN gamma signaling pathway	Biocarta			6.3×10^{-3}
IL 2 Signaling Pathway	Biocarta			4.3×10^{-3}
IL 3 Signaling Pathway	Biocarta			2.8×10^{-3}
IL 5 Signaling Pathway	Biocarta			1.9×10^{-6}
IL 6 signaling pathway	Biocarta		9.7×10^{-3}	
IL 7 Signal Transduction Pathway	Biocarta			7.3×10^{-3}
IL22 Soluble Receptor Signaling Pathway	Biocarta			8.0×10^{-3}

Lck and Fyn tyrosine kinases in initiation of TCR Activation pathway	Biocarta				4.3×10^{-3}
Multi-step Regulation of Transcription by Pitx2 pathway	Biocarta				6.0×10^{-3}
NO2-dependent IL 12 Pathway in NK cells pathway	Biocarta			8.5×10^{-3}	5.8×10^{-3}
Pelp1 Modulation of Estrogen Receptor Activity pathway	Biocarta				4.8×10^{-4}
RB Tumor Suppressor/Checkpoint Signaling in Response to DNA Damage Pathway	Biocarta				2.0×10^{-3}
Regulation of cell cycle progression by Plk3 pathway	Biocarta				7.0×10^{-4}
Role of Tob in T-cell activation pathway	Biocarta				5.0×10^{-3}
Sonic Hedgehog (SHH) Receptor Ptc1 Regulates cell cycle pathway	Biocarta				5.7×10^{-4}
Th1/Th2 Differentiation pathway	Biocarta		7.5×10^{-3}	7.9×10^{-3}	1.5×10^{-3}
The Co-Stimulatory Signal During T-cell Activation pathway	Biocarta				×
TNFR2 Signaling Pathway	Biocarta				3.4×10^{-4}
Adipogenesis Human	GenMapp	3.6×10^{-4}	5.8×10^{-3}	6.0×10^{-3}	0
Blood Clotting Cascade	GenMapp		9.2×10^{-3}		
Cell cycle	GenMapp			9.7×10^{-3}	0

Eicosanoid Synthesis	GenMapp		3.2×10^{-3}
Fatty Acid Omega Oxidation	GenMapp		
Inflammatory Response Pathway			2.1×10^{-3}
T Cell Receptor Signaling Pathway	GenMapp		1.4×10^{-4}
B Cell Receptor Signaling pathway	KEGG		2.0×10^{-3}
Cell cycle	KEGG		1.9×10^{-6}
Chronic Myeloid Leukemia	KEGG		7.4×10^{-3}
Gap junction	KEGG		3.5×10^{-4}
Jak-STAT signaling pathway	KEGG	8.7×10^{-3}	4.8×10^{-4}
Linoleic Acid Metabolism	KEGG		8.1×10^{-3}
Monoterpenoid biosynthesis	KEGG		3.0×10^{-3}
Natural Killer Cell Mediated Cytotoxicity	KEGG		4.8×10^{-3}
T cell receptor signaling pathway	KEGG		5.0×10^{-5}
Prostate cancer	KEGG	7.9×10^{-3}	9.8×10^{-5}

[a]Pathways that were identified as significant (estimated false-discovery rate < 0.01) following the removal of genes located in 6p21.3. The estimated false-discovery rate is listed in its respective cell.

[b]The threshold used as the probability of success.
